# Genome-wide association study reveals the genetic architecture of flowering time in rapeseed (*Brassica napus* L.)

**DOI:** 10.1093/dnares/dsv035

**Published:** 2015-12-10

**Authors:** Liping Xu, Kaining Hu, Zhenqian Zhang, Chunyun Guan, Song Chen, Wei Hua, Jiana Li, Jing Wen, Bin Yi, Jinxiong Shen, Chaozhi Ma, Jinxing Tu, Tingdong Fu

**Affiliations:** 1 National Key Laboratory of Crop Genetic Improvement, National Center of Rapeseed Improvement, Huazhong Agricultural University, Wuhan 430070, China; 2 College of Agronomy, Hunan Agricultural University, Changsha 410128, China; 3 Jiangsu Academy of Agricultural Science, Nanjing 210014, China; 4 The Oil Crops Research Institute, Chinese Academy of Agricultural Sciences, Wuhan 430062, China; 5 Chongqing Engineering Research Center for Rapeseed, College of Agronomy and Biotechnology, Southwest University, Chongqing 400716, China

**Keywords:** *Brassica napus*, flowering time, SNP, association mapping, linkage disequilibrium

## Abstract

Flowering time adaptation is a major breeding goal in the allopolyploid species *Brassica napus*. To investigate the genetic architecture of flowering time, a genome-wide association study (GWAS) of flowering time was conducted with a diversity panel comprising 523 *B. napus* cultivars and inbred lines grown in eight different environments. Genotyping was performed with a Brassica 60K Illumina Infinium SNP array. A total of 41 single-nucleotide polymorphisms (SNPs) distributed on 14 chromosomes were found to be associated with flowering time, and 12 SNPs located in the confidence intervals of quantitative trait loci (QTL) identified in previous researches based on linkage analyses. Twenty-five candidate genes were orthologous to *Arabidopsis thaliana* flowering genes. To further our understanding of the genetic factors influencing flowering time in different environments, GWAS was performed on two derived traits, environment sensitivity and temperature sensitivity. The most significant SNPs were found near Bn-scaff_16362_1-p380982, just 13 kb away from *BnaC09g41990D*, which is orthologous to *A. thaliana CONSTANS* (*CO*), an important gene in the photoperiod flowering pathway. These results provide new insights into the genetic control of flowering time in *B. napus* and indicate that GWAS is an effective method by which to reveal natural variations of complex traits in *B. napus*.

## 1. Introduction

Rapeseed (*Brassica napus* L., AACC, 2*n* = 38) is one of the most important oil crops in the world. It was grown on 36.5 million ha and produced 72.7 million tonnes of seed worldwide in 2013 (FAO 2013; http://faostat.fao.org/). Rapeseed is used not only as an edible oil but also as an industrial material for lubricants and biodiesel. *Brassica napus* is a recent allopolyploid species that evolved from natural hybridization between two diploid progenitor species, *Brassica rapa* (AA, 2*n* = 20) and *Brassica oleracea* (CC, 2*n* = 18), followed by chromosome doubling at least 10,000 yrs ago.^1,2^ It was domesticated as an oilseed crop only 400–500 yrs ago.^3,4^ According to the requirements of vernalization, *B. napus* can be divided into three different growth types: spring type, semi-winter type and winter type.

Flowering is an important transition from the vegetative stage to the reproductive stage, and correct timing of the floral transition is crucial to ensure reproductive success.^5^ Flowering time is a complex agronomic trait in *B. napus*. The potential of rapeseed yield depends to a large extent on flowering time.^6^ Rapeseed can be widely planted in China, which is the leading country for rapeseed production. One of the main reasons for its widespread growth is that it does not compete with cultivated land for summer crops such as rice and maize. The postponed flowering of rapeseed will affect summer crop planting; hence, flowering time adaptation is a major breeding goal. Currently, understanding of the genetics and molecular regulation of flowering time is mainly based on the model plant *Arabidopsis thaliana*. In *A. thaliana*, ∼180 genes have been identified for flowering time control.^7^ Five main flowering control pathways have been found.^5^ These five pathways are the vernalization pathway and the photoperiod pathway, which control flowering in response to seasonal changes in temperature and day length; the gibberellin pathway, which involves the requirement of gibberellic acid in flowering; the autonomous pathway, which is an endogenous regulation pathway that functions independently of the photoperiod and gibberellin pathways; and the endogenous pathway, which regulates flowering based on the age of the plant. The molecular mechanisms of these pathways have been studied extensively in *A. thaliana* and several other flowering plants.^5^

Previous studies of the genetic architecture of flowering time in rapeseed were based on QTL linkage mapping using traditional molecular markers, such as restriction fragment length polymorphisms, simple sequence repeats, intron polymorphisms and sequence-related amplified polymorphism markers in bi-parental populations. Many QTLs related to the flowering time of *B. napus* have been detected. A total of ∼15 QTLs with large phenotypic effects have been mapped, and these QTLs are mainly distributed on chromosomes A2, A9, A10, C2 and C3.^8–12^ Because *B. napus* and *A. thaliana* are cruciferous plants and close relatives, knowledge of genes related to flowering in *B. napus* is largely based on *A. thaliana* using comparative genomics. For example, *B. napus BnFLC1* is orthologous to *A. thaliana FLOWERING LOCUS C* (*FLC*), the key gene in the vernalization pathway,^13^ and *BnCOa1* is orthologous to *A. thaliana* CO, the core gene in the photoperiod pathway.^14^

Linkage mapping is restricted to allelic diversity and has limited genomic resolution.^15^ Genome-wide association studies, also called association mapping or linkage disequilibrium (LD) mapping, can be used to study millions of polymorphisms segregating in natural populations, which can be tested for their effects on a phenotype of interest.^16^ GWAS was first applied in the study of human diseases.^17^ With the development of sequencing technologies, GWAS has been successfully applied to the genetic dissection of complex traits in plants, such as *A. thaliana*,^18^ *Oryza sativa*,^19^ *Zea mays*^20^ and *B. napus*.^21,22^ Compared with linkage mapping, GWAS takes less research time, because it is not necessary to create mapping population, and it takes full advantage of ancient recombination events to identify the genetic loci underlying traits at a relatively high resolution.^23–25^

In this study, a diversity panel consisting of 523 *B. napus* cultivars and inbred lines was genotyped with the Brassica 60K Illumina Infinium SNP array. Flowering times of the panel were investigated in eight different environments. The objectives of this study were to (i) determine the extent of LD in the population, (ii) dissect the genetic architecture of flowering time in *B. napus* and (iii) investigate the genetic control of the variation in flowering time in different environments.

## 2. Materials and methods

### 2.1. Plant materials and field trials

A diversity panel, consisting of 523 *B. napus* cultivars and inbred lines, was used for association analysis in this study (Supplementary Table S1). These germplasms originated from 10 countries on 4 continents, and most came from China. These accessions included winter oilseed rape (OSR) (40), semi-winter OSR (433) and spring OSR (44) (Supplementary Table S1). The self-pollinated seeds for each accession were planted in the experimental field with a randomized complete block design of three replications in eight natural environments at five different locations. Each plot contained two rows, with 12 plants in each row and 20 cm between plants within each row and 30 cm between rows. They were sown at the end of September or the beginning of October and harvested the following May. The eight natural environments were E1 (Wuhan; 114.35°E, 30.48°N), E2 (Changsha; 113.09°E, 28.20°N), E3 (Nanjing; 119.18°E, 31.58°N), E4 (Ezhou; 114.90°E, 30.38°N) and E5 (Chongqing; 116.37°E, 40.017°N) in the 2012–13 growing seasons; and E6 (Wuhan), E7 (Chongqing) and E8 (Nanjing) in the 2013–14 growing season. The eight environments were semi-winter growing environment and the winter mean temperatures in the eight environments ranged from 0.8–7.7°C to 4.5–12.2°C (Supplementary Table S2).

### 2.2. Phenotyping and statistical analysis

Flowering time data were recorded as the number of days from the sowing day to the day 25% of the plants had at least one open flower in one plot in each environment. Flowering time was investigated for three replicates in E1, E5 and E6 and was investigated for only one replicate in the other five environments. Flowering time of each accession was defined as the average of the three replicates in the same environment. The coefficient of variation of flowering time (FT-CV) was used as a derived trait of environment sensitivity for each accession across the eight environments. FT-2013/2014 is the ratios of flowering times of the 2012–13 growing season to the 2013–14 growing season in the same location. SPSS software was used for the statistical analysis. The best linear unbiased prediction of flowering time (FT-BLUP) for each line in the eight environments was calculated using an R script (www.eXtension.org/pages/61006, 20 November 2015, date last accessed) based on a linear model.^26^

### 2.3. Genotyping and SNP marker filtering

Genomic DNA for genotyping was extracted from leaf tissues collected from three plants of each accession by a modified cetyltrimethylammonium bromide method.^27^ Genotyping was performed using the Brassica 60K Illumina Infinium SNP array. The SNP data were clustered and called automatically using Illumina BeadStudio genotyping software. The standards of quality control for SNP data were as follows: call frequency ≥0.8, minor allele frequency (MAF) ≥0.05 and homozygous genotype frequency cannot be zero. The probe sequences of the SNP array were used to perform a BLAST search against the *B. napus* Genomes Browser (http://www.genoscope.cns.fr/brassicanapus/, 20 November 2015, date last accessed). They were regarded as non-specific markers when BLAST matched to two or more locations in the reference genome. SNP markers used for LD analysis and association analysis were filtered with the following steps: (i) non-specific markers were excluded; (ii) markers that were not up to these standards of quality control for SNP data were eliminated; (iii) markers that did not have specific physical location information were excluded. SNPs that were selected for assessing population structure and relative kinship were filtered using the above first two steps.

### 2.4. Population structure, relative kinship and LD analysis

STRUCTURE 2.3.4 software was used to estimate the population structure with a Bayesian Markov Chain Monte Carlo model (MCMC). Each *K* value, as a putative number of populations set from 1 to 10, was obtained with five independent runs. The length of the burn-in period and number of MCMC replications after burn-in were set to 50,000 and 100,000, respectively. The true *k* value was determined by the log probability of the data (LnP(*K*)) and an *ad hoc* statistic Δ*K*, based on the rate of change in LnP(*K*) between successive *K* values.^28^ The Q matrix was the result of the integration of the cluster membership coefficient matrices of replicate runs from STRUCTURE by the CLUMPP software. Principal component analysis (PCA) was also used to assess the population structure. PowerMarker version 3.25 was used to calculate genetic distances among varieties by the method of Nei's genetic distances.^29^ After double centring, distance matrices were used to obtain eigenvectors in NTSYSpc version 2.1. The relative kinship matrix was calculated using SPAGeDi software, and all negative values between two individuals were set to 0. The parameter *r*^2^ was used to estimate LD by TASSEL version 4.0. The polymorphism information content (PIC) of the SNP markers was estimated using PowerMarker version 3.25.^30^

### 2.5. Genome-wide association analysis

Population structure and kinship were used to correct for false positives.^31^ The association analysis was performed with models by TASSEL version 4.0. The general linear model (GLM) included the naïve model that did not control for population structure and kinship; the Q model controlled the population structure using the Q matrix from STRUCTURE software to identify populations; the PCA model controlled the population structure using the top two principal components (PCs) from PCA to assess populations. The mixed linear model (MLM) included the following: the K model controlling kinship using the kinship matrix from SPAGeDi software to assess inter-individual relative kinship; the Q + K model controlling population structure with the Q matrix and kinship with the kinship matrix and the PCA + K model controlling population structure and kinship using the top two principal components and the kinship matrix, respectively. The Bonferroni test (0.05/number of tests) criterion is typically a very strict threshold;^32^ therefore, negative log (0.05/*n*) was used as a threshold for significance of associations between SNPs and traits, where *n* was the total number of SNPs used in the association analysis. In this study, the threshold was 5.6 (−log (0.05/21,117) ≈ 5.6). Quantile–quantile plots were created with a negative log value of the expected *P*-value from the genotype–phenotype association and the expected *P*-value from the assumption that no association exists between genotype and phenotype.

## 3. Results

### 3.1. Phenotypic variations

Flowering times of these accessions ranged from 94 to 202 days, with an average of 160 days across the eight environments, showing extensive variation in the association panel. Flowering times in different environments also showed great variation (Table 1 and Supplementary Fig. S1). The shortest average flowering times was 151 days in E5, and the longest was 168 days in E8 (Table 1). There were also differences in flowering times of the association panel at the same location in different years. For example, FT-2013/2014 is the ratio of flowering times across pairs of years in the same location. The average values of FT-2013/2014 were <1 in Changsha and Nanjing, and >1 in Wuhan (Table 1). This means that in Changsha and Nanjing, flowering times in 2013 were earlier than that in 2014. However, in Wuhan, flowering time in 2013 was slightly later than that in 2014. These results suggest that flowering time can be greatly affected by the environment.

**Table 1.** The descriptive statistics of phenotypic variations for flowering time in the association panel

Traits	Environment	Min	Max	Mean ± SE	Skewness	Kurtosis	CV (%)
FT (d)^a^	E1 (Wuhan-2013)	146	176	158 ± 0.17	0.98	2.29	2.48
	E2 (Changsha-2013)	152	190	160 ± 0.20	1.7	8.12	2.79
	E3 (Nanjing-2013)	147	182	161 ± 0.25	0.95	1.16	3.54
	E4 (Ezhou-2013)	128	202	163 ± 0.23	0.94	10.88	3.29
	E5 (Chongqing-2013)	102	183	151 ± 0.35	−0.09	3.98	5.23
	E6 (Wuhan-2014)	94	180	154 ± 0.42	−1.46	5.52	6.29
	E7 (Changsha-2014)	134	191	167 ± 0.24	−0.74	5.58	3.25
	E8 (Nanjing-2014)	151	189	168 ± 0.28	−0.32	0.4	3.80
FT-CV(%)^b^	E1–E8	1.85	15.38	4.12 ± 0.06	3.33	18.61	34.64
FT-2013/2014^c^	Nanjing	0.86	1.12	0.96 ± 0.00	0.93	1.99	3.13
	Wuhan	0.95	1.67	1.03 ± 0.00	4.75	37.09	6.05
	Changsha	0.9	1.13	0.96 ± 0.00	2.07	10.16	2.42

SE: standard error; CV: coefficient of variation.

^a^FT is the abbreviation of flowering time, which was recorded as the number of days from the sowing date to flowering.

^b^FT-CV is the coefficient variation of flowering time of each accession across the eight environments.

^c^FT-2013/2014 is the ratio of flowering times across pairs of years in the same location.

FT-CV ranged from 1.85 to 15.38%, with an average of 4.12%, and it had a CV of 34.64% (Table 1), showing extensive variation in the association panel. FT-CV, as a derived trait of environment sensitivity, was used to assess the stability of flowering time in different environments. The results show that flowering times of some accessions were stable in different environments and were not sensitive to environmental factors, whereas other accessions showed instability in flowering times under diverse environments.

### 3.2. Screening of SNPs and LD in rapeseed

**Figure 1.**
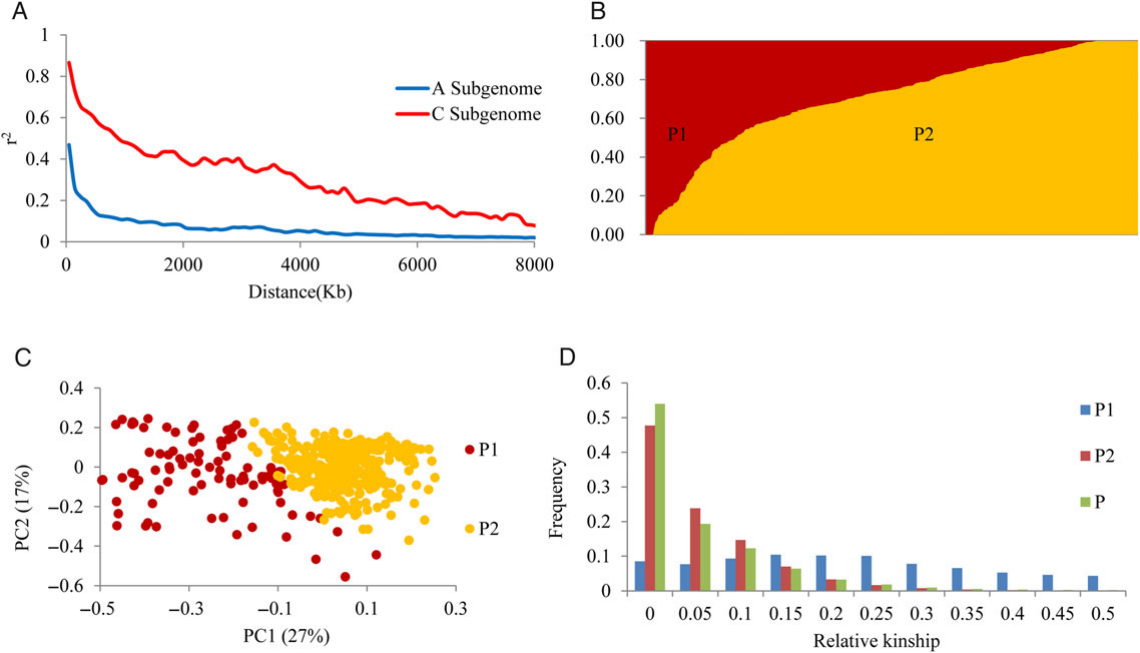
Analysis of linkage disequilibrium decay in two subgenomes and estimated population structure and relative kinships of the 523 rapeseed accessions. (A) Linkage disequilibrium decay determined by squared correlations of allele frequencies (*r*^2^) against distance between polymorphic sites in the A subgenome (blue) and C subgenome (red). (B) Population structure of 523 rapeseed accessions based on STRUCTURE when *K* = 2. (C) PCA of 523 rapeseed accessions; the top two principal components are illustrated in the bottom panels. (D) Distribution of pairwise relative kinship estimates in the entire population P (523 rapeseed accessions), subpopulation P1 and subpopulation P2. Only kinship values ranging from 0 to 0.5 are shown.

The Brassica 60K Illumina Infinium SNP array contained 52,157 SNPs. A total of 21,181 SNPs had BLAST matches to two or more locations in the reference genome and were therefore eliminated. In the remaining SNPs, 4,952 SNPs that were not up to the standards of quality control for SNP data were excluded. Finally, 26,024 SNPs were selected for assessing population structure and relative kinship. Of 26,024 SNPs, 21,117 had specific physical location information and were used for LD analysis and association analysis (Supplementary Table S3). These SNP markers were not evenly distributed across the whole genome. C9 had the lowest SNP marker density of one SNP per 77 kb, and A10 and C4 had the highest marker density of one SNP per 20 kb. The mean PIC values of the A subgenome and C subgenome were 0.3052 and 0.2974, respectively, and PIC values for each chromosome ranged from 0.2781 to 0.3219 (Table 2). LD was estimated as *r*^2^ (the squared pearson correlation coefficient) between all pairs of SNP markers. In the association panel, the LD decay of *B. napus* extending over a large distance was 6.5 Mb, where *r*^2^ = 0.1. The A subgenome and C subgenome were 1.2 and 7.8 Mb, respectively, and C subgenome LD was significantly higher than that of the A subgenome (Fig. 1A). LD decay ranged from 0.6 to 8.5 Mb among all chromosomes; however, the LD decay distance of chromosome A8 was 5.6 Mb, which was abnormally large compared with values for other chromosomes in the A subgenome (Table 2). These results revealed significant differences in the level of LD between different chromosomes and subgenomes.

**Table 2.** The summary of the number of SNPs mapped in each chromosome and the PIC and LD decay estimated for each chromosome

Chromosome	Number of SNPs	Density of SNP (kb/SNP)	PIC	LD decay (Mb)^a^
A1	855	27	0.3008	0.6
A2	699	35	0.3024	0.7
A3	1,185	25	0.3059	0.7
A4	894	22	0.2920	0.9
A5	901	26	0.3015	0.9
A6	821	30	0.3047	0.8
A7	1,064	22	0.3023	0.6
A8	589	31	0.2981	5.6
A9	906	37	0.3219	2.1
A10	885	20	0.3191	2.0
C1	1,855	21	0.2825	8.0
C2	1,663	28	0.3101	8.5
C3	1,992	30	0.3003	2.7
C4	2,417	20	0.3145	6.1
C5	631	68	0.3025	1.2
C6	940	39	0.2828	1.3
C7	1,152	37	0.2781	2.6
C8	1,050	36	0.2987	2.9
C9	618	77	0.2834	3.0

PIC: polymorphism information content.

^a^LD decay is the physical distance on the genome when the value of *r*^2^ is 0.1.

### 3.3. Population structure and relative kinship

Population structure was assessed by setting possible *K* values ranging from 1 to 10 with five replicates for each *K* value using STRUCTURE software. The LnP(*K*) value increased continuously with the increase of *K* from 1 to 10, and there was no obvious inflexion point. However, the most apparent change in LnP(*K*) appeared when *K* increased from 1 to 2, and the highest Δ*K* value was observed at *K* = 2 (Supplementary Fig. S2). Hence, the population could be divided into two clusters, P1 and P2 (Fig. 1B). Most lines originated from China, and only 47 lines were from other countries in the association panel. P1 contained 96 lines, of which 25 lines were from Europe and 2 lines were from Canada and Australia. P2 contained 424 lines, of which 10 lines were from Europe, 7 lines were from Japan and Korea and 4 lines had unknown origins. The others were all from China. Most of winter OSR germplasms and spring OSR germplasms were assigned into P2 and P1, respectively. (Supplementary Table S1). In addition, PCA was used to assess genetic variation in the panel. The top two PCs explained 44% of the genetic variation, and P1 and P2 formed clear clusters based on the two PCs (Fig. 1C).

The average relative kinship between any two inbred lines was 0.0443 in the panel; ∼54% of kinship estimates between lines were equal to 0, and 95% of kinship coefficients ranged from 0 to 0.2. The average pairwise relative kinship coefficient in P2 was 0.0423, and ∼96% of the kinship estimates between lines ranged from 0 to 0.2. The average pairwise relative kinship coefficient of P1, however, was 0.2652, which was greater than the values for the total population and P2. In P1, 49% of the pairwise relative kinship coefficients ranged from 0.2 to 0.5 (Fig. 1D). These results reveal that most lines in the panel and P2 had very weak kinship, and there was close kinship in the lines of P1.

### 3.4. Association mapping

**Figure 2.**
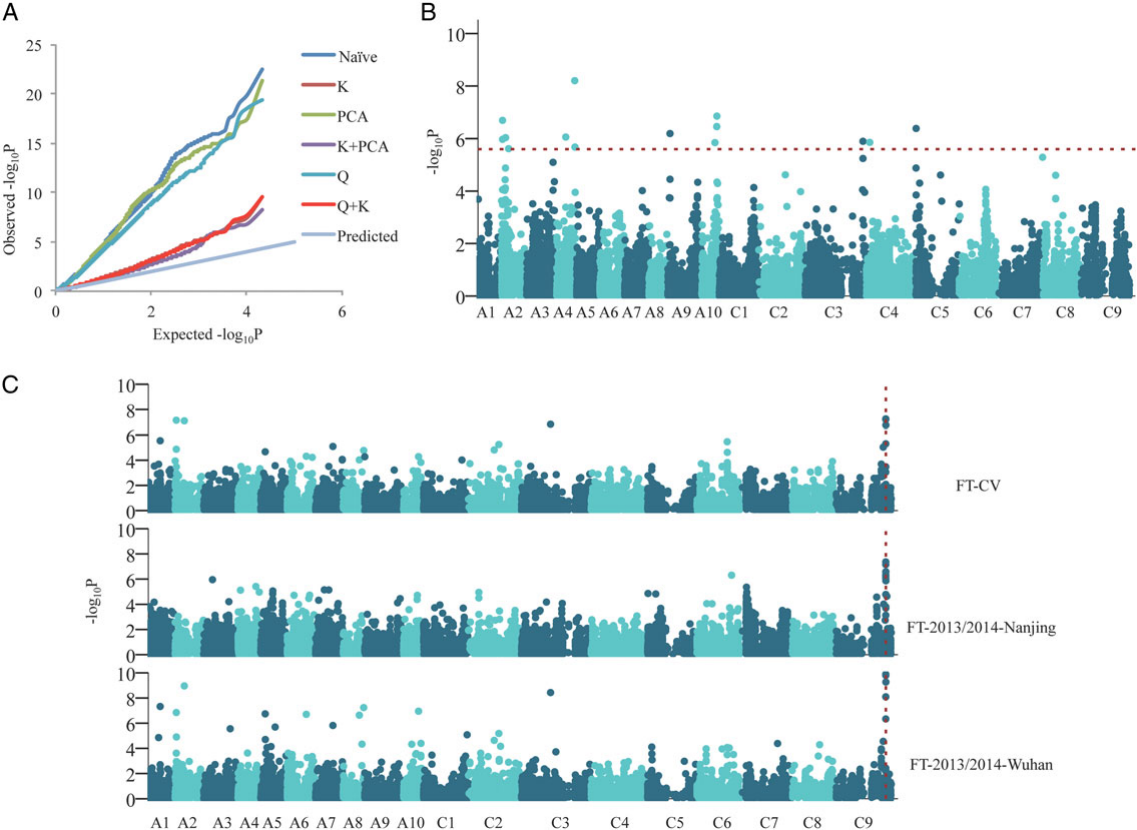
Genome-wide association scan for flowering time. (A) Quantile–quantile plots for flowering time using six models. (B) Manhattan plot for flowering time using the BLUP value. The dashed horizontal line represents the Bonferroni-adjusted significance threshold (*P* < 10^−5.6^). (C) Manhattan plot for environment sensitivity and temperature sensitivity. FT-CV as the trait of environment sensitivity was the coefficient of variation of flowering time of each accession across eight environments, FT-2013/2014-Nanjing and FT-2013/2014-Wuhan as the trait of temperature sensitivity were the ratios of flowering times across pairs of years in Nanjing and Wuhan, respectively. The common significant association signal Bn-scaff_16362_1-p380982 is outlined with a vertical dashed line.

To determine which model was more suitable for association mapping in our analysis, FT-BLUP was used to perform the association analysis in six models separately. The six models were as follows: the GLM model, including a naïve model; the Q model; the PCA model, the MLM model, including a K model; the PCA + K model; and the Q + K model. PCA and Q were used to control population structure, and K was used to control kinship. Except naïve model, PCA, Q and K were applied in corresponding models. As seen in the QQ plots (Fig. 2A), observed values had serious deviations from the expected values with the GLM model, which means that there was a high risk of false positives. Observed values were close to the expected values with the MLM model. Compared with the GLM model, the MLM model could better control false positives. Using PCA to estimate population structure was better than using the Q model in controlling false positives. To further control false positives, the PCA + K model was eventually selected for association mapping.

A total of 41 significant association signals for flowering time were identified with *P* < 10^−5.6^ by the PCA + K model in a genome-wide scan. Among the 41 SNPs, 40 SNPs were identified using the phenotypic values in individual environments (Supplementary Fig. S3 and Table 3), and 14 SNPs were identified using BLUP values across eight environments (Fig. 2B and Table 3). In total, nine SNPs were detected in more than two environments (Table 3). There was strong LD between two SNPs when their *r*^2^ (the squared Pearson correlation coefficient) was >0.75. In our study, these SNPs were considered to be the same QTL. These significant SNPs constituted 35 QTLs, which were distributed on 14 chromosomes across the *B. napus* genome and explained 5.28–15.75% of the phenotypic variation (Table 3).

**Table 3.** The summary of SNPs significantly associated with flowering time

QTLs	SNPs	Chromosome	Site	−log_10_P	*R*^2^ (%)^a^	Environment
1	Bn-A02-p3539297	A2	974,215	5.97–8.00	5.53–7.50	E6, BLUP
	Bn-A02-p3542024	A2	976,931	5.86–6.69	5.47–6.34	E3, E5, E6, BLUP
2	Bn-A02-p6845953	A2	388,7807	5.67	5.28	E3
3	Bn-A02-p6917044	A2	3,967,172	5.99–6.04	5.76–5.74	E1, BLUP
4	Bn-A02-p10129605	A2	6,976,101	5.62–6.45	5.50–5.94	E1, E4, BLUP
5	Bn-A02-p16528486	A2	12,748,561	5.77	5.88	E7
6	Bn-A03-p9247798	A3	8,529,640	5.77	5.55	E4
7	Bn-A03-p20318892	A3	19,186,356	5.65	5.99	E4
8	Bn-A03-p20358050	A3	19,224,858	5.75	5.64	E4
9	Bn-A04-p313410	A4	257,233	6.18	5.67	E4
10	Bn-A04-p2456200	A4	2,140,387	5.65	6.21	E8
11	Bn-A04-p7666812	A4	8,998,572	5.66–8.82	5.94–8.74	E1, E2, E4, BlUP
12	Bn-A04-p17212731	A4	17,888,394	5.74–11.40	5.88–10.65	E1, E2, E3, E5, E6, BLUP
13	Bn-A04-p17490425	A4	18,132,634	5.68–6.99	5.40–6.65	E1, E5, BLUP
14	Bn-A05-p22804927	A5	20,878,805	15.43	15.75	E4
15	Bn-A07-p15698133	A7	17,602,434	5.95	5.73	E1
16	Bn-A09-p1761036	A9	2,176,096	6.20–7.58	6.42–7.51	E1, E2, E3, BLUP
17	Bn-A09-p4997670	A9	29,577,613	5.95	5.91	E8
18	Bn-A10-p13051361	A10	13,087,115	5.85–7.08	5.50–6.64	E6, BLUP
19	Bn-A10-p14914898	A10	14,852,827	6.46–7.16	5.87–6.60	E1, E2, E3, E7, BLUP
20	Bn-A10-p15022346	A10	14,967,647	5.66–8.43	5.51–7.83	E1, E2, E3, E4, E7, BLUP
21	Bn-A10-p15106056	A10	15,049,331	5.74	5.414	E2
22	Bn-scaff_20675_1-p32364	C1	9,754,972	5.72	5.5	E4
23	Bn-scaff_17515_1-p685449	C1	34,087,382	5.65	5.47	E1
24	Bn-scaff_18507_1-p889927	C2	26,548,393	5.74	6.36	E5
25	Bn-scaff_17109_1-p683400	C2	41,638,730	6.00	6.44	E2
26	Bn-scaff_20103_1-p82288	C3	25,300,789	6.24	6.1	E6
27	Bn-scaff_17119_1-p235432	C3	57,106,138	5.90–7.60	5.65–7.30	E1, E4, BLUP
	Bn-scaff_17119_1-p235536	C3	57,106,242	6.59	6.13	E1
28	Bn-scaff_23098_1-p232984	C3	58,743,694	6.95	6.5	E2
29	Bn-scaff_16534_1-p719214	C4	3,307,887	5.86	5.98	BLUP
30	Bn-scaff_23107_1-p158176	C5	167,988	5.90	5.39	E2
	Bn-scaff_23107_1-p181951	C5	201,155	5.79–7.16	5.42–6.64	E2, E8, BLUP
31	Bn-scaff_20901_1-p369010	C5	3,670,150	6.70	6.26	E6
	Bn-scaff_20901_1-p335873	C5	3,698,179	6.06	6.2	E6
32	Bn-scaff_15856_1-p80690	C5	42,866,085	6.05	5.52	E2
33	Bn-scaff_15743_1-p392984	C6	27,572,975	6.07	6.06	E2
34	Bn-scaff_22835_1-p631232	C9	11,104,553	5.66	5.38	E3
35	Bn-scaff_16362_1-p404058	C9	43,721,308	6.86	6.32	E6
	Bn-scaff_16362_1-p385614	C9	43,727,934	6.63	6.14	E6
	Bn-scaff_16362_1-p380982	C9	43,732,634	5.85	5.41	E6

^a^*R*^2^ is the percentage of phenotypic variance explained by SNP.

**Figure 3.**
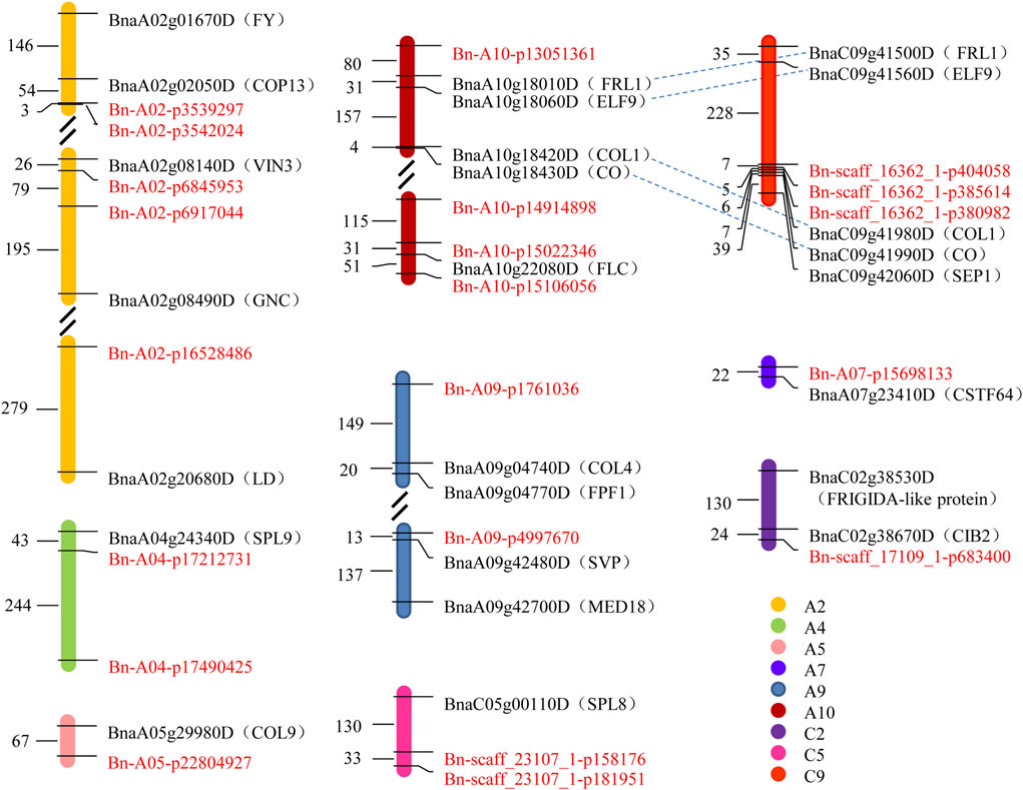
The distribution pattern of candidate genes and their corresponding SNPs associated with flowering time. The abbreviations of orthologous genes in *Arabptosis thaliana* are shown in brackets after the candidate genes. SNPs are marked in red. Homologous genes are connected by dashed lines. Numbers represent the relative distances in the genome, 1 = 1 kb.

Our research found that the LD decay of *B. napus* was in a relatively long distance (Table 2), and previous research showed that genome-wide significant associations for *B. napus* erucic acid content were, respectively, 233 and 128 kb away from the key genes *BnaA.FAE1* and *BnaC.FAE1*.^22^ In our study, *B. napus* genes orthologous to *A. thaliana* flowering time-related genes located within 300 kb of significant SNPs were treated as candidate genes. There were 25 candidate genes identified using this standard. The average *r*^2^ ranged from 0.1 to 0.85 in the regions between candidate genes and significant SNPs, and there were 15 candidate genes in high level of LD (*r*^2^ > 0.33) with corresponding SNPs associated with flowering time (Supplementary Table S4). The closest distance between the candidate genes and significant SNPs was ∼6 kb. Twelve genes were within 100 kb of their corresponding significant signals and included genes orthologous to *CO*, *FLC, VERNALIZATION INSENSITIVE 3*, and *SHORT VEGETATIVE PHASE* (Fig. 3). *Brassica napus BnaA10g22080D* which is orthologous to *A. thaliana FLC* was within 81 kb of two significant SNPs on A10, Bn-A10-p15022346 and Bn-A10-p15106056. There were four paralogous gene pairs in these candidate genes, which revealed that the QTL containing Bn-A10-p13051361 is homologous to the QTL that contain Bn-scaff_16362_1-p404058, Bn-scaff_16362_1-p385614 and Bn-scaff_16362_1-p380982 (Fig. 3 and Supplementary Table S4).

FT-CV, the CV of flowering times across the eight environments, as an environment sensitivity trait, and FT-2013/2014, the ratios of flowering times between 2 yrs at the same location, as the temperature sensitivity trait, were used to perform the association analysis. There were three groups of data on temperature sensitivity, and no significant signal (−log_10_P > 5.6) was found in Changsha. Twenty-four SNPs were found to be associated with temperature sensitivity, and six SNPs associated with environment sensitivity were also associated with temperature sensitivity. The most significant SNPs associated with environment sensitivity and temperature sensitivity were found near Bn-scaff_16362_1-p380982, located at 43.7 Mb of chromosome C9 (Fig. 2C and Supplementary Table S5). Bn-scaff_16362_1-p380982 was also associated with flowering time in E6 (Table 3). Three candidate genes, *BnaC09g41980D*, *BnaC09g41990D* and *BnaC09g42060D* that are, respectively, orthologous to *A. thaliana CONSTANS-like 1*, *CO* and *AGAMOUS-LIKE 2* were located in 52 kb away from the association signal (Fig. 3). These results reveal that there may be some important adaptation-related genes controlling flowering time near the association signal.

## 4. Discussion

### 4.1. LD in the rapeseed panel

In a population of 192 inbred lines of *B. napus*, the LD decayed within 0.5–1 cM.^33^ In another population of 472 rapeseed inbred lines, the longest LD decay was on chromosome C1 with 24,417 kb and the shortest was on chromosome A3 with 294 kb.^34^ In a diverse panel of 203 Chinese semi-winter rapeseed breeding lines, the LD decay of A subgenome and C subgenome was 0.25–0.30 and 2.00–2.50 Mb, respectively.^35^ Our study found that the LD decay was 6.5 Mb. Other species show LD decays of 100 kb–1 Mb (rice),^36^ 1–100 Kb (maize)^37–39^ and 250 kb (*A. thaliana*).^40^ These results reveal that the LD decay in *B. napus* is higher than that in other species, and the evolutionary time and mating system may be the main reasons for this difference. Mutation provides the raw material for producing polymorphisms that will be in LD, and recombination is the main phenomenon that weakens intrachromosomal LD.^41^ *Brassica napus* is a recent species that was formed ∼10,000 yrs ago.^1^ As such, there has been limited time for *B. napus* to accumulate more mutations and undergo restructuring events. Furthermore, *B. napus* is often cross-pollinated; fertilization events are approximately one-third cross-fertilization and two-thirds self-fertilization.^42^ Self-fertilization causes populations to become composed of homozygous individuals, and high levels of homozygosity limit the effectiveness of recombination.^43^ The comparatively high resolution provided by association mapping is dependent upon the structure of LD across the genome.^24^ The high level of LD may make it hard to get a high-resolution result by association mapping. In *B. napus*, GWAS has been proved to be an effective tool in fine mapping of complex traits in a population which has the level of LD similar to our population.^22^ However, the resolution provided by association study will be higher in the population with more diverse germplasm bases.

The average PIC value of the A subgenome was slightly higher than that of the C subgenome. This result is consistent with the finding that the A subgenome LD decayed in a relatively shorter distance compared with the C subgenome, meaning that the genetic diversity in the A subgenome is higher than that in the C genome. In our association panel, the majority of accessions were from China. Chinese *B. napus* has been improved by intentional introgression of genomic components from Chinese *B. rapa*.^44^ The A subgenome of *B. napus* is from European *B. rapa*, and Chinese *B. rapa* differs from European *B. rapa*.^1,44,45^ Therefore, the introgression into *B. napus* from Chinese *B. rapa* significantly increased the genetic diversity of the A genome. The LD of chromosome A8 decayed significantly slower than that of other chromosomes in the A subgenome. A similar result was found in previous studies.^34^ *BnaA.FAE1*, which has been cloned and confirmed as the key gene controlling the content of erucic acid in rapeseed, is on chromosome A8.^22,46,47^ The strong artificial selection in the process of breeding is probably an important cause of longer LD decay distances on chromosome A8.

### 4.2. Population structure and controlling false-positive results

This association population was classified into two subpopulations, but they were not completely separated according to geographical origin. In previous rapeseed association mapping panels, it is also hard to completely separate rapeseed lines according to geographical origin.^22,33^ Inter-specific crosses and hybridization between diverse germplasms from different countries were used to improve adaptation to the local environment and improve quality after *B. napus* spread to Australia, Canada, Japan and China.^48–50^ This spread resulted in introgression between germplasms of different countries. Moreover, there has been limited time for *B. napus* to undergo domestication after it was spread to different countries. Therefore, it is difficult to completely classify the association population according to geographical origins.

The Brassica 60K Illumina Infinium SNP array was developed by an international consortium preferentially using single-locus SNPs identified from genomic and transcriptomic sequencing in genetically diverse *Brassica* germplasm.^51^ This approach was designed before the *B. napus* ‘Darmor-Bzh’ genome^52^ was released. *Brassica napus* is an allotetraploid species, with some SNPs from homologous sequence variants (HSVs) and paralogous sequence variants (PSVs). Previous research has found that 87.5–91.2% of the polymorphisms were from homologous genes from the two subgenomes in *B. napus*.^53^ HSVs and PSVs will lead to genotyping errors of the association panel, which may cause false positives in subsequent association mapping. Hence, excluding SNPs not in a single MEGABLAST hit by BLAST analysis of probe sequences against the reference genome will eliminate interference from HSVs and PSVs.

Genotype–phenotype covariance can lead to spurious associations.^54^ Many genetic markers appear to be associated with phenotype. In fact, these genetic markers simply capture the genetic relatedness among individuals.^55^ Flowering time was particularly strongly correlated with geographic origins and population structure in *Z. mays* and *A. thaliana*.^56,57^ The trait and polymorphism(s) were associated very strongly when population structure was ignored, but the association disappeared when structure was considered.^57^ As described in Fig. 2A, compared with the naïve model, the models making use of Q and PCA to control false positives from the population structure slightly reduced inflation of *P*-values. The MLM model, including the K model, PCA + K model and Q + K model, can markedly reduce inflation of *P*-values. Therefore, *P*-values were serious inflated when kinship was ignored. This indicates that relative kinship within the population probably is one of the major factors resulting in false positives in our association study.

### 4.3. QTLs and candidate genes for flowering time involvement

Twelve SNPs associated with flowering time were located in,^9,10,12,58–60^ and seven SNPs near,^9,10^ the confidence intervals of QTLs identified in previous studies based on linkage analysis (Supplementary Table S6). There were 11 SNPs allele frequencies that showed significant difference between spring OSR group and winter OSR group (Supplementary Table S7). These SNPs corresponding candidate genes included some important vernalization-related orthologous genes, for example, *BnaA10g22080D*, *BnaA02g08140D* and *BnaC09g41500D* (Fig. 3 and Supplementary Table S7). *BnaA10g22080D* was the only candidate gene in the 300 kb range of the three SNPs Bn-A10-p14914898, Bn-A10-p15022346 and Bn-A10-p15106056, and was in the same location as *BnFLC.A10*.^61^ There was only one amino acid difference in the amino acid sequences of *BnaA10g22080D* and *BnFLC1*, and expression of *BnFLC1* in *A. thaliana* delayed flowering significantly relative to untransformed L*er*.^13^ *BnaA10g22080D* is likely a functional allele related to vernalization. In addition, the remaining SNPs that have not been identified previously in linkage analysis may be novel and important QTLs related to flowering time; they should be verified by other methods in future research.

Flowering time is under the control of diverse environmental stimuli, such as temperature and photoperiod.^5^ The temperature and photoperiod of different regions have significant differences, and there are also significant differences between the temperatures at the same location in different years. Considering the influence of environmental factors on flowering, the dates of flowering time in the eight different environments were separately analysed with association mapping. BLUP minimized the impacts of different locations and years on flowering time, and conversely, the derived trait of environment sensitivity highlighted these impacts. In the same location, the average temperatures of 2013–14 growing season were higher than that of 2012–13 growing season (Supplementary Table S2). Therefore, the ratio of flowering times between 2 yrs in the same location was regarded as a derived trait of temperature sensitivity. Six SNPs associated with environment sensitivity were also associated with temperature sensitivity (Fig. 2C and Supplementary Table S5). This result suggests that a particular factor affecting flowering times in different years in the same location may have been the major factor that led to differences in the flowering times in the eight environments. Temperature is one of the major environmental factors, but other factors affecting the flowering time could not be ruled out and should be studied in future research. Deep insight into the genetic factors and environmental factors influencing flowering time in different germplasms has a large application potential in rapeseed breeding and cultivation. The *BnaC09g41990D* orthologue of *A. thaliana CO* is only 13 kb away from the common significant association signal of environment sensitivity and temperature sensitivity. The temporal and spatial regulation of CO is key to the photoperiod-dependent induction of flowering, and recent research has found that CO protein stability is also affected by temperature.^5,62^ The TARGET OF EAT1 (TOE1) proteins interact with the activation domain of CO proteins and inhibit CO activity,^63^ and expression of *TOE1* was reduced at 23°C compared with levels at 16°C.^64^ CO degradation by *HIGH EXPRESSION OF OSMOTICALLY RESPONSIVE GENE 1* is increased under low temperature conditions (4°C).^65,66^ *BnCO*a1, *BnCO*a9, *BnCO*b1 and *BnCO*b9 are orthologous to the Arabidopsis *CO* gene and were isolated from a pair of homoeologous loci in each of two *B. napus* lines displaying different flowering times. *BnCOa1* has been shown to rescue the *co-2* mutation in Arabidopsis.^14^ BLAST analysis showed that BnaA10g18430D and BnCOa9, and BnaC09g41990D and BnCOb1 have the same amino acid sequences. BnaA10g18430D and BnaC09g41990D were shown to have 98.7 and 94.3% identity, respectively, to BnCOa1 at the amino acid level. Given the above data, *BnaC09g41990D* and *BnaA10g18430D* are important candidate genes that may control *B. napus* flowering time using photoperiod and temperature.

## Supplementary data

Supplementary data are available at www.dnaresearch.oxfordjournals.org.

## Funding

This work was supported by the National High Technology Research and Development Program of China (Grant No. 2012AA101107), 948 Program of Ministry of Agriculture of China (2011-G23), the National Basic Research Program of China (2011CB109300) and Natural science Foundation of Hubei province Key program (2014CFA008). Funding to pay the Open Access publication charges for this article was provided by the National High Technology Research and Development Program of China (Grant No. 2012AA101107).

## Supplementary Material

Supplementary Data
