# Supplementary material for: Genome-wide association study reveals the genetic architecture of flowering time in rapeseed (Brassica napus L.)
Source: DNA Res. 2015 Dec 10;23(1):43–52. doi: 10.1093/dnares/dsv035 (PMC4755526; doi:10.1093/dnares/dsv035)
Supplement: Supplementary Data [file supp_23_1_43__index.html]

Genome-wide association study reveals the genetic architecture of flowering time in rapeseed (Brassica napus L.) — Genome-wide association study reveals the genetic architecture of flowering time in rapeseed (Brassica napus L.) — Supplementary Data 

# Genome-wide association study reveals the genetic architecture of flowering time in rapeseed (*Brassica napus* L.)

## Supplementary Data

Supplementary Data

- Supplementary Figures - pdf file
- Supplementary Table 1 - xls file
- Supplementary Table 2 - xls file
- Supplementary Table 3-1 - xlsx file
- Supplementary Table 3-2 - xlsx file
- Supplementary Table 3-3 - xlsx file
- Supplementary Table 4 - xls file
- Supplementary Table 5 - xls file
- Supplementary Table 6 - xls file
- Supplementary Table 7 - xls file
