# Supplementary material for: Genome-wide association study reveals the genetic architecture of flowering time in rapeseed (Brassica napus L.)
Source: DNA Res. 2015 Dec 10;23(1):43–52. doi: 10.1093/dnares/dsv035 (PMC4755526; doi:10.1093/dnares/dsv035)
Supplement: Supplementary Data [file supp_dsv035_dsv035supp_figures.pdf]

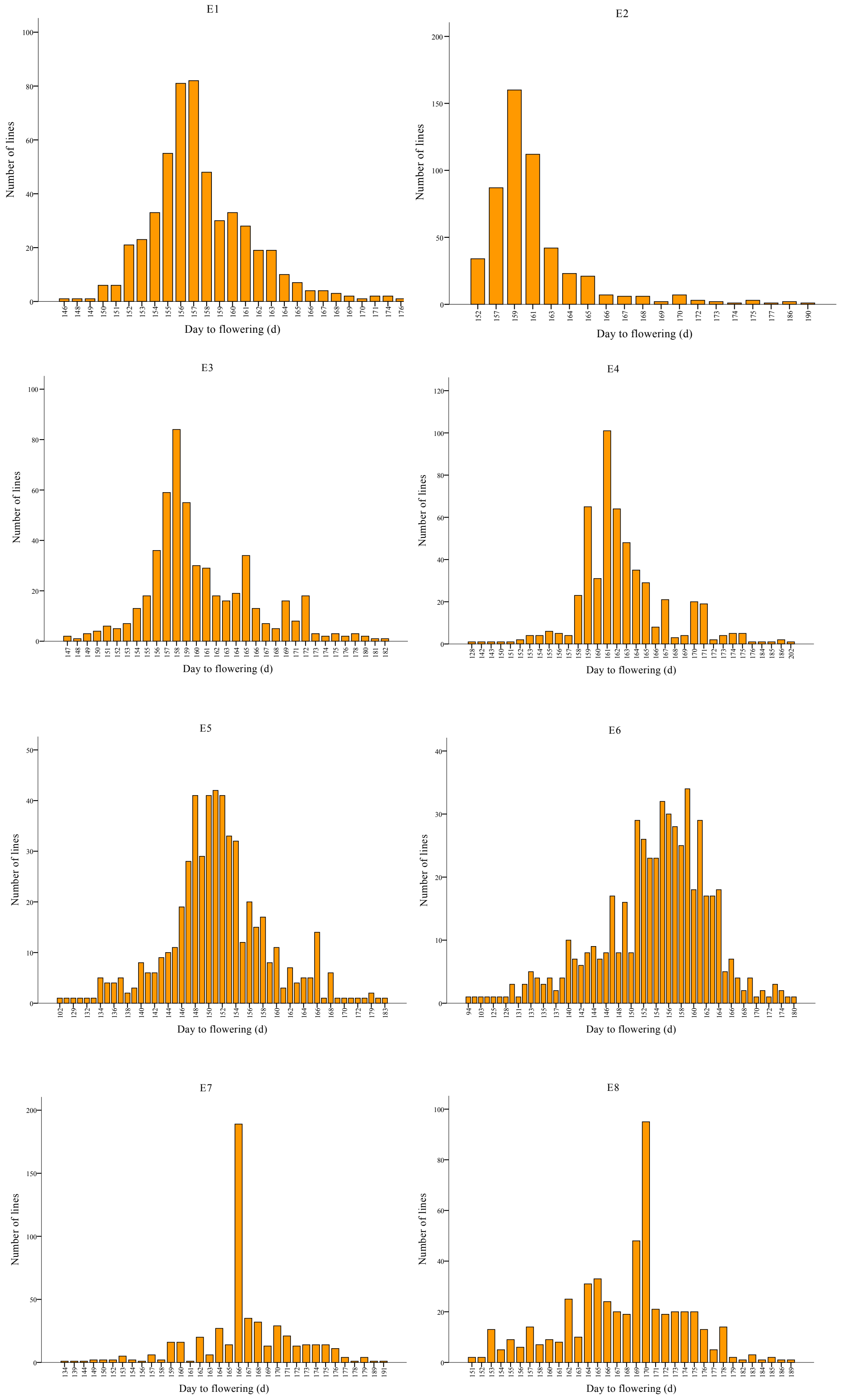

Supplementary Figure S1. Distributions of flowering times in eight environments.

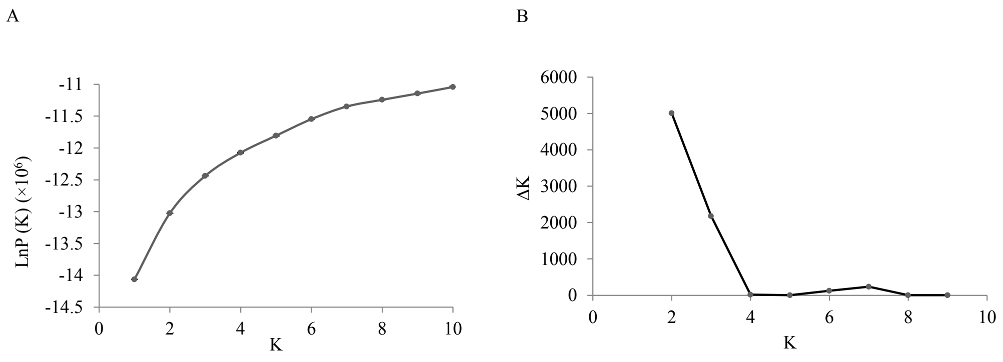

Supplementary Figure S2. Estimation of LnP(K) and ΔK in the association panel.

(A) Estimated LnP(K) of possible clusters (K) from 1 to 10.

(B) ΔK based on the rate of change of LnP(K) between successive K values.

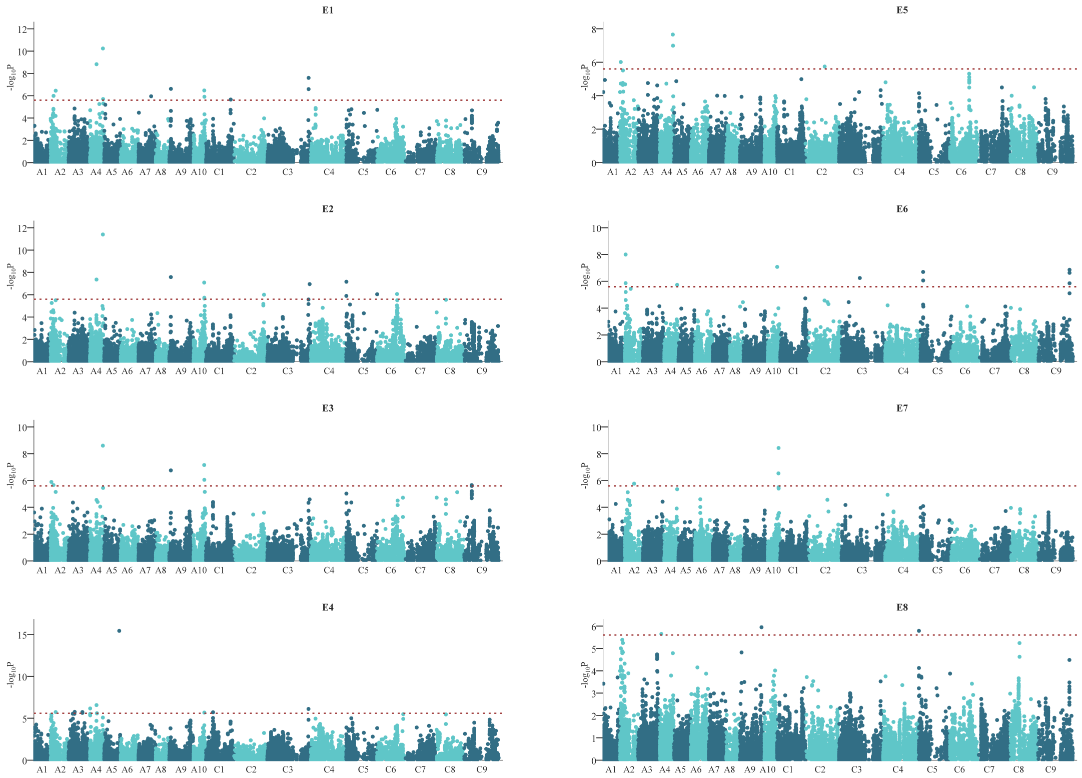

Supplementary Figure S3. GWAS results for flowering times in eight environments.

The dashed horizontal line represents the Bonferroni-adjusted significance threshold ($P < 10^{-5.6}$).
